# Ectomycorrhizal Colonization and Diversity in Relation to Tree Biomass and Nutrition in a Plantation of Transgenic Poplars with Modified Lignin Biosynthesis

**DOI:** 10.1371/journal.pone.0059207

**Published:** 2013-03-13

**Authors:** Lara Danielsen, Gertrud Lohaus, Anke Sirrenberg, Petr Karlovsky, Catherine Bastien, Gilles Pilate, Andrea Polle

**Affiliations:** 1 Department of Forest Botany and Tree Physiology, Büsgen-Institute, Georg-August University of Göttingen, Göttingen, Germany; 2 Department of Molecular Phytopathology and Mycotoxin Research, University of Göttingen, Göttingen, Germany; 3 INRA, UR0588 Amélioration, Génétique et Physiologie Forestières, CS 40001 Ardon, Orléans, France; Brigham Young University, United States of America

## Abstract

Wood from biomass plantations with fast growing tree species such as poplars can be used as an alternative feedstock for production of biofuels. To facilitate utilization of lignocellulose for saccharification, transgenic poplars with modified or reduced lignin contents may be useful. However, the potential impact of poplars modified in the lignification pathway on ectomycorrhizal (EM) fungi, which play important roles for plant nutrition, is not known. The goal of this study was to investigate EM colonization and community composition in relation to biomass and nutrient status in wildtype (WT, *Populus tremula* × *Populus alba*) and transgenic poplar lines with suppressed activities of cinnamyl alcohol dehydrogenase, caffeate/5-hydroxyferulate O-methyltransferase, and cinnamoyl-CoA reductase in a biomass plantation. In different one-year-old poplar lines EM colonization varied from 58% to 86%, but the EM community composition of WT and transgenic poplars were indistinguishable. After two years, the colonization rate of all lines was increased to about 100%, but separation of EM communities between distinct transgenic poplar genotypes was observed. The differentiation of the EM assemblages was similar to that found between different genotypes of commercial clones of *Populus* × *euramericana*. The transgenic poplars exhibited significant growth and nutrient element differences in wood, with generally higher nutrient accumulation in stems of genotypes with lower than in those with higher biomass. A general linear mixed model simulated biomass of one-year-old poplar stems with high accuracy (adjusted R^2^ = 97%) by two factors: EM colonization and inverse wood N concentration. These results imply a link between N allocation and EM colonization, which may be crucial for wood production in the establishment phase of poplar biomass plantations. Our data further support that multiple poplar genotypes regardless whether generated by transgenic approaches or conventional breeding increase the variation in EM community composition in biomass plantations.

## Introduction

The growing world population inevitably entails an increasing energy demand along with diminishing fossil fuel resources [1]. Renewable energies from biomass can be used as an alternative to partially replace conventional energy supplies. Trees, especially fast-growing species such as poplars, are an appealing feedstock for this purpose because they can be grown in dense short rotation plantations allowing several harvests without the need to re-plant [2]. Furthermore, poplars have a low nitrogen demand compared with other potential bioenergy crops [3]. Thus, their cultivation may contribute to the mitigation of nitrogen emissions from intensely used agricultural areas [4].

The conversion process of biomass to biofuels requires the breakdown of plant cell walls, which mainly consist of cellulose, hemicelluloses, and lignin [5]. Lignin is a recalcitrant polymer composed of phenylpropanoid units that hinder chemical and enzymatic cellulose degradation necessary for bioethanol production [6]. To amend wood utilization cell wall properties have been changed by targeted genetic approaches [7]. Genes of the biosynthetic pathway of lignin and cellulose have been isolated and characterized [8]–[10]. Suppression of cinnamyl alcohol dehydrogenase (CAD), an enzyme which converts cinnamyl aldehydes to the respective alcohols [5] and caffeate/5-hydroxyferulate O-methyltransferase (COMT), an enzyme involved in biosynthesis of syringyl lignin [5] result in altered lignin composition compared to wildtype (WT) poplars [11]–[13]. Overexpression of ferulate 5-hydroxylase (F5H), an enzyme that catalyzes an intermediate step in lignin biosynthesis, also results in compositional changes and less polymerization of monolignol units compared to the WT [14]. Suppression of cinnamoyl-CoA reductase (CCR) causes reduced lignin contents [15]. Transgenic poplars with alterations in lignin content and composition have been tested for industrial usage and display improved Kraft pulping [16]. The saccharification efficiency is also increased by genetic engineering of the lignin biosynthetic pathway [17].

If the use of genetically modified (GM) poplars with improved wood properties for bioenergy production was expanded, it will be necessary to know whether nutrient status and ecological interactions of GM poplars are changed compared with the WT. In a preceding study we compared whole fungal communities in soil and roots of poplars with suppressed CAD activities and of the WT by pyrosequencing and found a strong dominance of ectomycorrhizal (EM) in roots, whereas saprophytes were prevalent in soil [18]; significant differences of these traits between the CAD lines and WT were not found [18]. The interaction of poplar roots with EM fungi is of particular importance for nutrient acquisition [19]. But other benefits have also been reported such as higher survival rates of EM-inoculated young poplar saplings [20]–[23] and increased resistance to drought stress [24]–[26], issues gaining importance with increasing poplar cultivation in a warming climate. Currently it is still unclear if changes in the lignification pathway have significant ecological implication for interacting organisms. Lignin is the end product of the phenylpropanoid pathway, whose modification generally has consequences for the biosynthesis of other phenol-bearing compounds. For example, the suppression of CCR results in decreased lignin, but increased concentrations of phenolic compounds [15]. Phenolic compounds have been implicated in a wide range of ecological interactions. Greenhouse studies have shown that enzymatic activities of microbial communities are altered in soil of poplars with reduced lignin concentrations [27]. Field studies on the EM communities in relation to the performance of poplars with changes in the lignin composition and reduction of the lignin concentrations are lacking.

The aim of this study was to characterize the EM community composition and dynamics in the first cycle of a short rotation plantation with poplars modified in the lignification pathway. To assess the relationship between EM diversity, plant nutrient status and dendromass we analyzed height growth, biomass, and nutrient element composition in leaves, stem and roots of transgenic *Populus × canescens* with suppressed activities of COMT (L9 and L11), CCR (L5 and L7), or CAD (L18, L21 and L22) and the wildtype (WT). We further compared the EM assemblages in the GM plantation with those of commercial poplar clones (*P*. x *euramericana*, syn, *Populus deltoides* × *Populus nigra* c.v. Ghoy, I-214, and Soligo). Our study shows that in the first year after plantation establishment, EM fungal colonization and diversity were linked with tree productivity and low stem nitrogen concentrations. The variation of the EM fungal community composition found on roots of different transgenic poplar genotypes was similar to that found on different commercial poplar genotypes.

## Materials and Methods

### Plant material and field site

One hybrid clone of *Populus tremula* × *Populus alba* (INRA #717-1B4, syn. *P. × canescens*) referred to as wild type (WT) and seven transgenic lines from this WT clone modified in key enzymes of the lignin biosynthetic pathway were used to establish a field trial. The transgenic lines were down regulated in one of the following enzymes of the lignin biosynthesis pathway: CCR (cinnamoyl coenzyme A reductase) with line FS3 = L5 and FAS13 = L7 [15], COMT (caffeic acid O-methyl transferase) with line ASOMTB2B = L9 and ASOMTB10B = L11 [11], and CAD (cinnamyl alcohol dehydrogenase) with line ASCAD21 = L21, ASCAD52 = L18, and SCAD1 = L22 [28]. After multiplying the clones by micropropagation [29] 120 plants of each of the 8 different poplar lines were planted in a plowed area of 1365 m^2^ on sandy soil with flint in June 2008, next to INRA in Orléans, Sologne, France (47°83′N, 1°91′E). The field trial with GM poplars with modified lignin (application B/FR/07/06/01) has been approved by the “Bureau de la réglementation alimentaire et des Biotechnologies” from the “Direction Générale de l”Alimentation” from the French “Ministère de l”Agriculture et de la Pêche” (ministerial decision #07/015 on September 21, 2007 for a 5 year period). The land, where the field trial was conducted, is owned by INRA. Protected species were not sampled.

In this area the mean annual temperature is 10.4°C and precipitation 600 mm. The plant density was chosen according to short rotation coppice practice as follows: the space between trees of one double row was 0.55 m while the interspace between the two double rows was 1.5 m, and the planting distance within a line was 1 m (Fig. S1). The poplar lines were planted in a randomized block design with 5 blocks. Each block consisted of eight plots, one for each line. Each plot consisted of 24 trees (4×6) planted in two double rows. To prevent edge effects the experimental plantation was bordered with one row of WT clones (Fig. S1). During the growing season the poplars were drip irrigated.

A second plantation with 11 commercial clones of *Populus deltoides × P. nigra* including the cultivars Blanc de Poitou, Carpaccio, Dorskamp, Flevo, Ghoy, I-214, Koster, Lambro, Robusta, Soligo, and Triplo was established in May 2009 in the same area. The random block design consisted of three blocks. Each block consisted of 11 plots. Each plot consisted of 16 trees (4×4) of one commercial clone. The space between trees of one double row was 0.6 m while the interspace between the two double rows was 1.5 m, and planting distance within a line was 0.6 m (Fig. S2)

### Sampling of soil cores for analyses of roots and soil

Soil cores were harvested immediately after planting (July 2008) to assess the heterogeneity of soil fungi and nitrogen at the beginning. After plantation establishment soil were collected for ECM fungal community analysis in October 2009 and October 2010. In July 2008, 25 soil cores (diameter: 8 cm, depth: 20 cm) were taken randomly in the experimental field, the border area, and the area between the experimental field and a nearby poplar plantation.

In October 2009 and 2010 three plots per clone (i.e. 1 WT +7 GM lines) were randomly chosen and soil cores (diameter: 5 cm, depth: 20 cm) were collected within these plots. Three trees per plot were chosen and three soil cores per tree were taken at a distance of 0.25 m from the trunk. In total 27 soil cores per line were collected. Soil cores were transported on ice and stored at 4°C until further processing.

Sampling in the *P. deltoides* × *P. nigra* plantation took place in October 2010, one year after planting. The same sampling strategy was used for the plantation with the commercial poplar clones as described above for the transgenic poplars. Three clones were selected for the analysis based on growth differences, which were mainly caused by differences in *Melampsora larici-populina* leaf rust infection: Soligo (high growth and high rust resistance), Ghoy (low growth and low rust resistance) and I-214 (intermediate growth and intermediate rust resistance).

### Fungal soil communities analyzed by denaturing gradient gel electrophoresis (DGGE)

DGGE was performed for fungal soil communities at the time point of GM plantation establishment (June 2008). Twenty-five soil samples were sieved and 250 mg sieved soil was used for DNA isolation with the PowerSoil™ DNA Isolation Kit (MO BIO Laboratories, Inc., Canada). The primer pair ITS1 and ITS4 [30] was used to amplify the rDNA ITS-region of fungi. A GC-clamp was added to the 5′ end of the ITS4 primer to stabilize the melting behavior of the Polymerase Chain Reaction (PCR) products in the gel according to Muyzer *et al*. [31].

PCR was performed according to the following protocol: the total volume of the reaction mix was 25 µl, containing 2 µl template DNA, 2 µl of MgCl_2_ (25 mM) (Fermentas, St. Leon-Rot, Germany), 2.5 µl 10× buffer (Fermentas, St. Leon-Rot, Germany), 1.25 µl of each primer (stock: 10 µM) (Eurofins MWG Operon, Ebersberg, Germany), 0.5 µl dNTPs mix (10 mM each, Fermentas, St. Leon-Rot, Germany), 15.375 µl of nuclease-free water, and 0.125 µl *Taq* polymerase (>10 U/ µl, Fermentas, St. Leon-Rot, Germany). A Master Cycler (Eppendorf, Hamburg, Germany) was used to amplify the DNA with the following cycle steps: hot-start at 95°C for 15 min, followed by 95°C for 1 min, 34 cycles of 30 s at 94°C (denaturation), 30 s at 55°C (annealing) and 1 min at 72°C (extension), and termination at 72°C for 5 min.

The separation of the rDNA sequences was achieved in a 7.5% polyacrylamide (37.5: 1 =  acrylamide: bis-acrylamide) gel with a linear denaturing gradient from 32–65% of denaturant (100% denaturant containing 40% (v/v) formamide and 7 M urea). After 2 h of polymerization 7.5 ml of 7.5% polyacrylamide gel without denaturant was added (stacking gel). After 20 min of polymerization the gel was loaded with 4 µl of PCR product per lane of each of the 25 samples. Running buffer contained 0.5× TAE (20 mM tris(hydroxymethyl)-aminomethane, pH 7.4, 10 mM sodium acetate, 0.5 mM disodium ethylenedinitrilo-tetraacetic acid). An INGENYphorU-2 system (Ingeny International, Goes, The Netherlands) was used for the DGGE at a constant temperature of 58°C, 120 V and a running time of 16 h. DNA bands were visualized by silver staining following the “SILVER SEQUENCE™” protocol (Promega Corporation, Madison, USA). The stained gels were scanned on a flat-bed scanner. The band patterns were manually converted into a present/absent matrix, which was subjected to similarity analyses (Table S1).

### Free amino acids, nitrate and ammonium in soil samples

At the time point of plantation establishment (June 2008), the concentrations of nitrogen compounds (nitrate, ammonium, amino acids) in the soil solution were determined. Soil samples were sieved (mesh width 5 mm) and 40 g of fresh soil were mixed with 40 ml 1 mM CaCl_2_, incubated for 10 min and filtered through a Whatman® folded filter (Ø185 mm, Ref.No. 10314747, Whatmann, Dassel, Germany). After 1 h the resulting filtrate was passed through a glass fiber filter (pore size 1 µm, Pall Life Science, Port Washington, NY, USA) and subsequently through a sterilization filter (0.2 µm Sarstedt Filtropur S, Nümbrecht, Germany). After volume determination, the filtrate was freeze-dried and dissolved in 0.5 ml double deionized H_2_O. Amino acids were analyzed by high-performance liquid chromatography (Pharmacia/LBK, Freiburg, Germany) according to Tilsner et al. [32]. Nitrate and ammonium were determined by photometric measurements (Shimadzu UV 1602, Hannover, Germany) using enzymatic ammonium and nitrate test kits (Merck 100683, Merck 109713, Merck, Darmstadt, Germany). The concentrations of inorganic nitrogen and amino acids are reported in Table S2.

### Ectomycorrhizal colonization and morphotyping

For the investigation of the EM fungal community of roots, soil cores were divided longitudinally, and the three samples, which had been collected around the stem of one tree, were pooled resulting in nine samples per poplar line. Roots were carefully separated from the soil by washing in a sieve under running tap water. The washed roots were inspected under a stereomicroscope (M205 FA, Leica, Wetzlar, Germany) and non-poplar roots were removed from the sample. The root samples were weighed and aliquots were removed, dried and used for nutrient element analyses.

Subsequently, living and dead root tips were counted until a total number of 300 living roots tips per sample was reached. The numbers of the different morphotypes and of the dead root tips were recorded applying a simplified method after Agerer [33]. Dead root tips exhibited a shrunken and dry appearance. EM morphotypes were distinguished by color, shape, texture of the mantle, and absence or presence of rhizomorphes and/or hyphae. Samples of each morphotype were collected and stored at −20°C for molecular analysis.

EM colonization (%) was calculated as: EM root tips ×100/(EM root tips + vital non-mycorrhizal root tips).

The vitality index of root tips was determined as: number of living root tips ×100/total number of counted root tips.

### Sanger sequencing of the fungal ITS region

For the extraction of genomic DNA of frozen EM root tips the “innuPREP Plant DNA kit” (Analytik jena, Jena, Germany) was used following the instructions of the manufacturer. The primer pair ITS4 and ITS5 [30] was used to amplify the rDNA ITS-region by PCR with the PCR protocol described above for the DGGE. Cloning and sequencing or direct sequencing were conducted according to Druebert et al. [34]. The following databases were used for nucleotide BLAST searches: UNITE (http://unite.ut.ee/), Fungal RSyst (http://mycor.nancy.inra.fr/RSyst/), and NCBI BLASTn (http://www.ncbi.nih.gov/). Fungal sequences have been deposited at NCBI with the accession numbers JQ409279 to JQ409296 and JQ824878 to JQ824884, respectively.

### Stem heights and biomass

Heights of trees chosen for EM fungal analysis were measured in October 2009 and 2010, respectively, when seasonal growth had stopped. In 2010 in addition to the height (h) of the leader shoot the number and lengths of side shoots, and stem diameters (d) of all shoots (15 cm above ground) were measured. Fully expanded top leaves were collected (Oct 2009) and dried for nutrient analyses.

Trees were coppiced in March 2010 and above ground stem biomass was determined after drying at 40° for two weeks to constant weight. Since there is no growth between October and March (fall/winter season), the stem biomass data measured in March 2010 represent that of the preceding year (2009).

Biomass in October 2010 was calculated as: V • ρ with V = 1/3 • r^2^ • π • h where r = d/2 and ρ = 0.50 g • cm^−^^3^ [35], [36].

### Nutrient element and δ^13^C analyses

Dry stem wood (March 2010), roots (October 2010) and leaves (October 2010) were cut into small pieces, mixed and aliquots were removed and milled to a fine powder (MM2, Retsch, Hannover, Germany). Nutrient elements were pressure-extracted in HNO_3_ and measured by inductively coupled plasma optical emission spectrometry (ICP-OES) after Heinrichs *et al*. [37]. For N and C analyses powdered dry tissues were weighed into tin cartridges (Hekatech, Wegberg, Germany) and measured with an element analyzer (Element Analyzer EA-1108, Carlo, Erba Instruments, Rodano, Italy). Leaf and wood samples for δ^13^C analysis were weighed into tin cartridges (Hekatech, Wegberg, Germany) and analyzed with an isotope mass spectrometer (Delta plus XP, Finnigan MAT, Bremen, Germany) coupled with an element analyzer (EuroVektor, HEKAtech GmbH, Wegberg, Germany).

### Statistical analyses

Statistical analyses were conducted using R statistics version 2.9.2 [38]. To identify potential clusters in the distribution of soil fungi (detected by DGGE) and soil nutrients (soluble amino acids, nitrate, and ammonium) across the plots non metric multidimensional scaling (NMDS) was conducted with package: “vegan” [39]. Input parameters were Jaccard distance for soil fungi and Euclidean distance for soil nutrients, respectively. To find out if the soil fungal assemblages were related to the composition of the soluble nitrogen compounds in soil, data were subjected to a Mantel test with the package “vegan” [39].

Similarities of EM fungal community structures in 2009 and 2010 were analyzed by NMDS using Bray-Curtis distance as input parameter. In all cases a maximum of 100 starts were used to find a stable solution. The procedure was repeated with the best previous solution to prevent local optima. Function envfit() was used to fit grouping factors (different lines) onto the ordination. 95% confidence ellipses were drawn with function ordiellipse(), package:”vegan” [39].

Data for height, biomass, mycorrhizal colonization, vitality index, nutrient element concentrations and δ ^13^C signature are shown as means (±SE). Significant differences at p ≤ 0.05 were detected by one-way ANOVA followed by multiple comparisons with TukeyHSD (package: “stats”). Residuals of the models were analyzed by Kolmogorov-Smirnov and Levene”s test to check for normal distribution and homogeneity of variances, respectively. If one of the assumptions of the ANOVA had to be rejected, Kruskal-Wallis rank sum test followed by Mann Whitney U test was conducted.

Rarefied diversity indices (Shannon-Wiener Index (H′), species richness, and Pielou”s Evenness) based on 850 root tips per sample were calculated using the EcoSim software version 7.72 [40]. Since cumulative rarefied diversity indices for the EM fungi community were calculated, only one value per line and year was obtained. Regression analysis and general mixed models (GLM) were calculated with Statgraphics Centurion (StatPoint Technologies, Inc.,Warrenton, VA). Residuals of the regression models were tested by Shapiro Wilks normality test to check the assumption of normal distribution. If the assumption of normal distribution had to be rejected the Null Hypothesis that the slope is equal to zero was tested by Spearman”s rank correlation. Before starting the analysis the data were checked graphically for outliers followed by Dixon test for outliers, package: “outliers” [41].

## Results

### Absence of fungal clusters and nutrient patches in the soil of a poplar plantation

**Figure 1:**
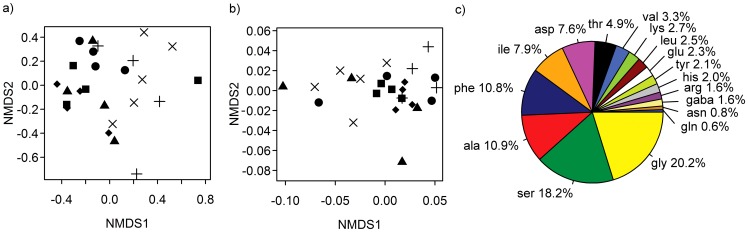
Non metric multidimensional scaling (NMDS) of soil parameters in a poplar (*P*. x *canescens*) plantation. (a) Fungal communities: The soil fungal pattern was determined by DGGE and similarities determined as Jaccard distances were used for the NMDS analysis (two of four dimension are shown, stress = 9.72). (b) Soluble nitrogen compounds in the soil solution: NMDS of sum of free amino acids, nitrate, ammonium (two of three dimensions are shown, stress = 5.91). For the analysis 25 soil samples were used collected at the positions marked in Figure S1. The samples were annotated to their location in the plantation: upper part (filled diamond), upper-middle (filled square), middle-bottom (filled triangle), bottom (filled circle) and outside as border area (+) and distant area (X). (c) Amino acids in the soil solution: Mean percentage of soluble amino acids of all samples. Ser: serine, asn: asparagine, glu: glutamic acid, asp: aspartic acid, lys: lysine, leu: leucine, phe: phenylalanine, ile: isoleucine, val: valine, tyr: tyrosine, gaba: gamma-aminobutyric acid, ala: alanine, arg: arginine, thr: threonine, gly: glycine, gln: glutamine, his: histidine. Measurements were conducted when the plantation was installed (2008).

When the poplar plantation was established in June 2008, nitrogen in the soil solution and fungal distribution were determined to detect potential patchy distribution of soil nutrients and fungi. NMDS did neither reveal any clustering for the patterns of soil fungi (Fig. 1a, permutation test, R^2^ = 0.30, p = 0.144) nor for soluble nitrogen in the soil solution at different sampling spots in the plantation (Fig. 1b, R^2^ = 0.34, p = 0.101). Other soil nutrient elements and soil pH neither showed positional effects [18].

The mean concentration of the sum amino acids was 415±38 nmol kg^−1^ soil. Glycine, alanine, serine, phenylalanine and isoleucine were the most abundant amino acids in the soil (Fig. 1c). The mean soil concentrations of inorganic nitrogen were 82.6±7.0 µmol kg^−1^ for nitrate and 16.6±0.9 µmol kg^−1^ for ammonium. To test if the concentrations of the soluble nitrogen compounds in the soil were correlated with the fungal distribution a Mantel test was conducted. No correlation of those parameters was found (r = −0.065, p = 0.634). Since we did not detect clustering of soil fungi or nutrient patches when the plantation was established it is unlikely that further results were influenced by local variations of these environmental factors.

### Ectomycorrhizal colonization show temporal dynamics and genotype- but not gene-specific effects in GM poplars

One year after planting (2009) the EM colonization varied between the different transgenic poplar lines and WT from 58% to 86% (Table 1). CAD line L22 showed the lowest and CAD line L18 the highest colonization (Table 1). At the end of the following growing season (2010) almost all vital root tips were colonized with EM (Table 1). There was only very little variation between the lines (Table 1).

**Table 1:** Ectomycorrhizal (EM) colonization, vitality index and root density of *P. × canescens*.

	EM colonization [%]		Vitality index [%]		Root density [g l ^−1^]	
	2009	2010	2009	2010	2009	2010
	F = 2.1939	F = 1.1465	F = 2.3565	F = 1.9684	F = 6.783	F = 0.9578
	p = 0.04758*	p = 0.3462	p = 0.0342	p = 0.0735	p<0.001	p = 0.4697
WT	71±5.4 a	99±0.4 a	85±4.9 ab	96±1.2 a	0.503±0.168 bc	0.962±0.321 a
CCR L5	64±7.3 a	99±0.6 a	89±5.1 ab	98±0.6 a	0.543±0.205 c	0.896±0.299 a
CCR L7	73±10.2 a	100±0.0 a	79±5.9 ab	92±2.3 a	0.104±0.039 a	0.739±0.246 a
COMT L9	82±4.8 a	99±0.4 a	76±5.2 ab	95±1.5 a	0.133±0.047 ab	0.652±0.217 a
COMT L11	75±4.1 a	100±0.1 a	91±2.5 a	94±1.9 a	0.384±0.128 c	0.862±0.287 a
CAD L18	86±1.7 a	99±0.3 a	86±5.0 ab	96±1.3 a	0.497±0.166 c	0.774±0.258 a
CAD L21	64±5.9 a	100±0.2 a	91±2.3 ab	97±1.1 a	0.447±0.149 c	1.146±0.382 a
CAD L22	58±8.2 a	99±0.4 a	67±8.8 b	91±2.8 a	0.256±0.090 abc	0.689±0.230 a

Root density was determined as root mass per liter of soil volume. Significant differences are indicated by different letters (ANOVA, followed by TukeyHSD, p≤0.05). Values indicate mean ± SE, (n = 7–9). CCR, COMT and CAD refer to transgenic poplar lines with suppressed activities of cinnamoyl coenzyme A reductase, caffeic acid O-methyl transferase, and cinnamyl alcohol dehydrogenase, respectively. *no significant differences were detected by TukeyHSD.

**Figure 2:**
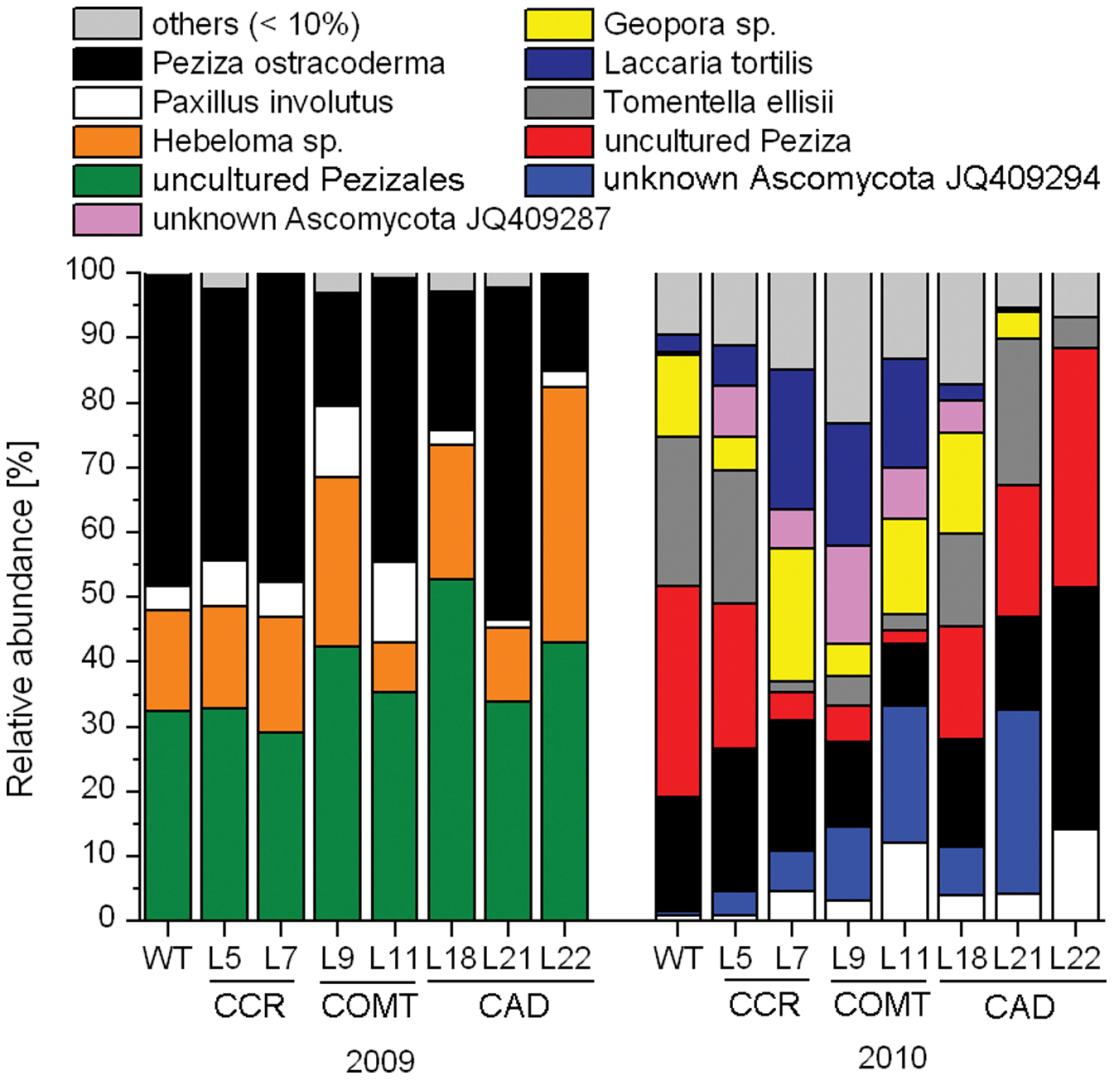
Relative abundance of the most frequent ectomycorrhizal species on the roots of wildtype (WT) and transgenic *Populus × canescens* genotypes. The plantation was established in June 2008 and ectomycorrhizal (EM) colonization were determined in October 2009 and October 2010. Only those EM species are shown that exceed on average at least 10% colonization in one host line, other detected species are summarized as “others”. Different colours represent different ECM species. The complete species list is found in Table S3. CCR, COMT and CAD refer to transgenic poplar lines with suppressed activities of cinnamoyl coenzyme A reductase, caffeic acid O-methyl transferase, and cinnamylalcohol dehydrogenase, respectively.

The higher EM colonization of roots after two years than after one was also accompanied by higher EM species richness: only eight different EM species were detected after one, however, 30 after two years (Fig. 2, Table S3). Of the 30 EM species, six (*Paxillus involutus, Laccaria tortilis, Hebeloma sacchariolens, Hebeloma* sp., *Cenococcum geophilum* and *Peziza ostracoderma*) had already been present in the preceding year (Table S3). The increases in total ECM species numbers were also reflected in the Shannon-Wiener Index, which increased from a mean across all poplar lines of 1.2 in 2009 to 2.1 in 2010 (p<0.001), the Simpson Index, which increased from 0.65 to 0.83 (p<0.001), and rarefied species richness, which increased from 5.5 to 13.6 (p<0.001), whereas Evenness was unaffected (mean 2009: 0.72, mean 2010: 0.78, p = 0.22, Table S4). It was striking that CAD line L22 showed for all diversity indices one of the lowest and COMT line L9 generally the highest values, especially in the first year after plantation. CAD line 22 also displayed higher root tip mortality in 2009 than the other poplar genotypes, whereas its root density assumed an intermediate position between CCR line L5 (highest) and CCR line L 7 (lowest, Table 1).

**Figure 3:**
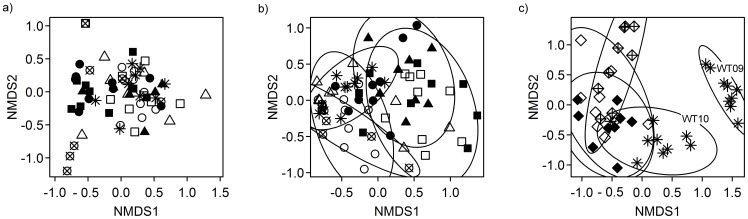
Non metric multidimensional scaling (NMDS) of the ectomycorrhizal communities associated with transgenic and commercial poplar genotypes. (a) NMDS of EM communities of wildtype and transgenic *P*. x *canescens* in 2009. Two of three dimensions are shown (stress = 10.20, permutation test for separation R^2^ = 0.49, p = 0.073). (b) NMDS of EM communities of wildtype and transgenic *P*. x *canescens* in 2010. Two of four dimensions are shown (stress = 11.70, permutation test for separation R^2^ = 0.43, p = 0.001). (c) NMDS of EM communities of three *P. deltoides × P. nigra* clones (2010) and the wildtype of *P.× canescens* in 2009 and 2010. Two of four dimension are shown (stress = 7.80, permutation test for separation R^2^ = 0.76, p = 0.001). Symbols correspond to different poplar lines. (a,b) COMT: open (L11) and filled squares (L9), CCR: open (L5) and filled triangles (L7), CAD: open (L21), filled (L18) and crossed circles (L22) and WT: star. (c) *P. deltoides × P. nigra* clones: open (Ghoy), filled (I-214) and crossed diamonds (Soligo), *P*. x *canescens*: stars.

To investigate potential genotype-related effects on EM associations, we analyzed the EM community composition in greater detail. One year after plantation establishment, four of the total number of eight detected EM species were dominant colonizing >90% of the mycorrhizal root tips of all poplar lines; no significant differences between CAD, CCR, COMT and WT lines were found (Fig. 2). NMDS of the ECM fungal community on 1-year-old poplars neither revealed significant separation of different poplar lines (permutation test R^2^ = 0.1649, p = 0.073, Fig. 3a).

At the end of the second year (2010), eight EM species were relatively frequent on the root tips (>10% colonization per EM species) with some significant differences between the poplars lines (Fig. 2): an uncultured *Peziza* was more abundant on WT than on CCR line L7 and COMT line L11 roots (Mann-Whitney U-Test, p = 0.022 and p = 0.031, respectively). *Laccaria tortilis* was more abundant on COMT line L11 than on CAD lines L21 and CAD line L22 (p = 0.0077 and p = 0.0087, respectively) (Fig. 2). The changes in fungal abundance and composition resulted in genotype-related shifts in the EM communities as documented by NMDS (Fig. 3b, permutation test R^2^ = 0.43, p = 0.001). The transgenic lines CCR L7 and CAD L22 showed a complete separation of their EM community structures (Fig. 3b). CAD line L18, CCR line L5 and COMT line L9 showed the strongest overlap (for clearness of display ellipses not drawn). The EM community structure of the WT was overlapping with all other lines indicating no significant separation (Fig. 3b).

To elucidate the ecological importance of these observations we also analyzed the EM species composition of three genotypes of high-yielding, commercial clones of *P*. x *euramericana* (Ghoy, I-214, and Soligo) in comparison with the WT of *P. × canescens*. The ordination shows a clear separation of the EM communities of one- and two-year-old *P. × canescens* (permutation test: R^2^ = 0.76, p = 0.001, Fig. 3c). Among the three *P*. x *euramericana* clones studied Ghoy and I-214 showed overlapping EM communities with *P. × canescens*, whereas Soligo was almost completely separated from *P. × canescens* and had less overlap with Ghoy and I-214 than those two genotypes among each other (Fig. 3c). These results support that the EM communities underlie temporal and genotype-specific differentiation. However, a separation of EM communities according to the modification of lignification genes was not found.

### Early genotype-specific variation of growth is related to stem N concentrations and ectomycorrhizal root colonization

Since EM fungi can affect nutrient uptake and plant performance, we investigated growth and nutrient status of the poplars in the GM plantation. Significant differences were found for height growth and biomass among the poplar genotypes (Table 2). CAD line L22 generally exhibited the lowest performance and CAD line L18 the best (Table 2). CAD line L18 also produced more side shoots than the other poplar genotypes (Table 2). In comparison with the WT the lines CAD L22 and CCR L7 showed reduced biomass production, whereas biomass of the other genotypes was unaffected by the genetic modification (Table 2).

**Table 2:** Growth and biomass of wildtype (WT) and transgenic *P. × canescens* genotypes.

	Height (cm)	Height (cm)	Cum height (cm)	Biomass (g)	Biomass* (g)	Shoots no.	RCD (mm)
	2009	2010	2010	2009	2010	2010	2010
	F = 5.349	F = 9.9638	F = 5.129	F = 3.291	F = 7.9226	F = 2.862	F = 5.101
	p<0.001***	p<0.001***	p<0.001***	p = 0.006**	p<0.001***	p = 0.012*	p<0.001
WT	205.1±11.2 ac	322.7±10.5 c	1100.6±135.1 bc	132.6±13.0 a	417.2±32.9 c	5.6±0.9 ab	23.8±1.4 a
CCR L5	185.4±18.8 abc	304.6±12.1 ac	847.6±103.8 abc	88.0±18.3 ab	247.8±51.3 ac	4.4±0.6 ab	20.0±1.4 ab
CCR L7	154.6±15.5 ab	239.7±19.3 ab	639.3±117.8 ab	79.8±18.1 ab	179.1±46.9 ab	3.3±0.3 a	16.7±1.7 ab
COMT L9	203.6±6.9 ac	309.9±22.4 ac	786.7± 84.8 ab	139.1±12.7 a	330.0±58.6 ac	3.8±0.5 ab	21.5±1.7 a
COMT L11	216.6±15.5 c	305.6±14.0 ac	837.8±121.0 abc	121.8±16.5 ab	302.1±43.8 ac	4.3±0.8 ab	19.6±2.2 ab
CAD L18	224.2±13.9 c	328.3±15.4 c	1310.0±103.8 c	130.5±14.7 a	466.9±65.9 c	6.7±0.6 b	24.1±1.8 a
CAD L21	220.1±9.8 c	343.1±4.6 c	970.4±102.5 abc	118.0±18.8 ab	371.4±42.8 c	4.4±0.6 ab	22.0±1.9 a
CAD L22	137.8±7.4 b	194.2±9.1 b	505.2±63.2 a	33.4±4.1 b	77.5±11.7 b	3.7±0.5 ab	±0.4 b

CCR, COMT and CAD refer to transgenic poplar lines with suppressed activities of cinnamoyl coenzyme A reductase, caffeic acid O-methyl transferase, and cinnamyl alcohol dehydrogenase, respectively. The plantation was established in June 2008 and measurements were taken in October 2009 and October 2010. Data are means (± SE, n = 9). Cum Height: cumulated height of all stems of one plant was calculated as the sum of the length of the main stem and the side shoots. Biomass  =  dry mass of the main stem, RCD: root collar diameter. Significant differences are indicated by different letters (ANOVA, followed by TukeyHSD p≤0.05). *  =  calculated with estimated stem volumes and wood density.

To find out whether the growth differences of the different poplar genotypes were the results of compromised nutrient supply, the nutrient element status was characterized for leaves, wood and stem, and carbon allocation was assessed by analyses of the δ^13^C signatures in leaves and stem biomass (Table S5). The mean δ^13^C value of leaves was −27.34±0.11‰ and that of stems −24.92±0.03‰ (p<0.001). This indicates differences in carbon discrimination between leaves and stem; but no genotype-related effects within leaves or stems were found. We have, therefore, no evidence that the growth differences were caused by genotype-related differences in photosynthetic carbon allocation to wood.

The nutrient element concentrations did not reveal nutritional deficits in comparison with other poplars [42], but significant differences between the analyzed poplar genotypes were detected (Table 3, Table S5). The highest number of differences in nutrient element concentrations among the genotypes was found in stems (P, N, K, Mg, Ca, Mn), an intermediate number in leaves (P, N, K, C, S) and the lowest number of differences were found in roots (P, K, Mn). These results indicate genotype-specific differences in internal nutrient element allocation. The macronutrients P and K showed genotype-related effects in all tissues and N in leaves and stems. The latter three nutrient elements were analyzed in greater detail since their uptake is known to be regulated by EM fungal associations [19].

**Table 3:** P, N and K concentrations in stems of wildtype and transgenic poplar (*P. × canescens*).

Tissue	Genotype	P (mg/g)				N [mg/g]				K [mg/g]			
Leaves	WT	2.832	±	0.170	ab	25.479	±	0.898	abc	11.544	±	0.307	abc
Leaves	CCR L5	3.021	±	0.092	ab	28.486	±	0.700	a	11.901	±	0.299	ab
Leaves	CCR L7	2.616	±	0.124	ab	23.632	±	0.783	b	10.615	±	0.271	ac
Leaves	COMT L9	2.776	±	0.179	ab	25.488	±	0.389	abc	12.273	±	0.471	ab
Leaves	COMT L11	3.184	±	0.178	a	26.492	±	0.561	abc	12.049	±	0.532	ab
Leaves	CAD L18	2.749	±	0.059	ab	25.053	±	0.734	bc	12.613	±	0.517	b
Leaves	CAD L21	3.169	±	0.139	a	28.169	±	1.150	ac	12.552	±	0.442	b
Leaves	CAD L22	2.461	±	0.063	b	23.890	±	0.518	b	9.926	±	0.339	c
Leaves	All	F = 3.72				F = 5.47				F = 5.54			
Leaves	All	P = 0.002				p<0.0001				p<0.0001			
Stem	WT	1.139	±	0.021	b	8.226	±	0.314	bd	2.653	±	0.032	c
Stem	CCR L5	1.221	±	0.052	ab	9.222	±	0.315	ab	3.352	±	0.152	a
Stem	CCR L7	1.318	±	0.055	ab	9.881	±	0.222	ac	3.422	±	0.173	a
Stem	COMT L9	1.215	±	0.053	ab	8.204	±	0.229	bd	2.813	±	0.122	bc
Stem	COMT L11	1.250	±	0.029	ab	8.197	±	0.197	bd	2.963	±	0.078	abc
Stem	CAD L18	NA				NA				NA			
Stem	CAD L21	1.330	±	0.050	a	8.055	±	0.210	d	2.772	±	0.086	bc
Stem	CAD L22	1.370	±	0.056	a	10.757	±	0.263	c	3.245	±	0.122	ab
Stem	All	F = 2.83				F = 13.62				F = 7.18			
Stem	All	p = 0.019				p<0.0001				p<0.0001			
Roots	WT	1.561	±	0.071	ab	8.717	±	1.104	a	5.369	±	0.238	ab
Roots	CCR L5	1.825	±	0.066	abc	9.444	±	1.103	a	5.834	±	0.249	ab
Roots	CCR L7	1.760	±	0.094	ab	9.794	±	0.618	a	6.036	±	0.357	ab
Roots	COMT L9	1.618	±	0.094	ab	10.185	±	0.538	a	5.542	±	0.306	ab
Roots	COMT L11	1.499	±	0.030	a	10.283	±	0.969	a	5.502	±	0.286	ab
Roots	CAD L18	1.931	±	0.111	bc	11.201	±	0.949	a	6.054	±	0.438	ab
Roots	CAD L21	2.156	±	0.094	c	10.169	±	1.106	a	6.672	±	0.399	a
Roots	CAD L22	1.609	±	0.088	ab	9.555	±	1.070	a	5.063	±	0.330	b
Roots	All	F = 6.87				F = 0.59				F = 2.23			
Roots	All	p<0.0001				p = 0.760				p = 0.043			

CCR, COMT and CAD refer to transgenic poplar lines with suppressed activities of cinnamoyl coenzyme A reductase, caffeic acid O-methyl transferase, and cinnamyl alcohol dehydrogenase, respectively. F statistics and p-values are given for one-way ANOVA (p≤0.05). Significant differences between poplar lines are indicated by different letters. Data indicate means ± SE (L22: n = 4, all other n = 7–9). NA  =  not available.

Multiple variable analyses revealed no significant correlations of the P concentrations in any of the analyzed tissues with EM-related parameters such as root colonization, EM species richness, the Shannon Wiener index or root tip vitality (Table S6). To find out if the P concentrations were related to the abundance of specific EM fungi, i.e., related to fungal identity, multiple variable analyses were carried out for the dominant fungi with the tissue nutrient concentrations. None of the nutrient elements (stem concentrations of P, K, or N) showed significant correlations with the abundance of any of the major EM fungi in 2009. In 2010, the leaf P and K concentrations were negatively correlated with the relative abundance of *Peziza ostracoderma* (for P: R = −0.808, p = 0.015; for K: R = −0.713, p = 0.047) and the leaf P concentrations were positively correlated with the abundance of an unknown ascomycete JQ409294 (R = 0.747, p = 0.033). Although leaf P concentrations were correlated with height (Table S6), a link between height and the abundance of the ascomycete JQ409294 could not be established (p = 0.19). Therefore, we have no evidence for interactions between distinct EM fungal species, P concentrations and growth.

**Figure 4:**
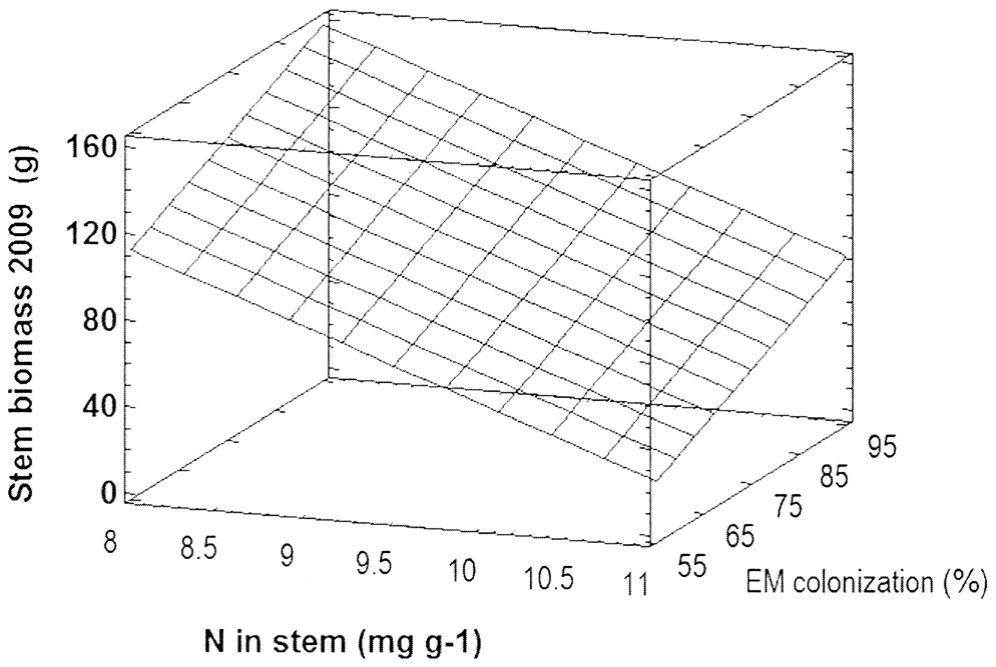
A general linear mixed model for stem biomass with stem N concentrations and root ectomycorrhizal colonization as quantitative independent factors. The surface (hatched lines) shows the 3-dimensional relationship between biomass, N concentration and mycorrhizal colonization.

To further evaluate the relationship between growth, tissue nutrient element concentrations and EM assemblages, we searched the correlation matrix for significant p values (Table S6). Stem biomass (2009) was significantly correlated with EM fungal species richness (2009), root tip colonization (2009), stem K and stem N concentrations. GLM analyses with these parameters and stepwise removal of the factor with the least significant P-value revealed that stem biomass (2009) was modeled with high accuracy by only two factors: stem N concentrations and mycorrhizal root colonization (adjusted R^2^ = 97%, F_(model)_ = 108.4, P_(model)_ = 0.0003, F_(N)_ = 101.1, P_(N)_ = 0.0006, F_(EM)_ = 10.8, P_(EM)_ = 0.03, Fig. 4). Stem biomass was negatively related to N concentrations and positively with the degree of EM root tip colonization (Fig. 4).

## Discussion

### Influence of gene modification on mycorrhizal colonization and community structure

Poplars can form mutualistic associations with both arbuscular mycorrhizal and EM fungi [19]. However, in poplar plantations associations with EM fungi are the dominant symbiotic form [18], [21]. Age-related increases in root tip colonization and EM species diversity as observed here for GM and WT poplars are well known for non-transgenic as well as transgenic poplars (e.g., suppression of the *rolC* gene in *P.* x *canescens* [43], wildtype *P. tremuloides* [44]). Besides the dynamic fungal succession, we observed initially differences in root tip colonization, which vanished in the second year and a differentiation of distinct EM communities on different poplar genotypes.

A main question of the current study, therefore, was if the changes in EM colonization and fungal species composition were caused by the suppression of genes of the lignification pathway. Decreases in lignin as caused by CCR suppression or changes in the lignin composition as caused by CAD and COMT suppression interfere with secondary metabolism and entail changes in the profiles of phenolic compounds [45]. Since phenolic compounds belong to the defense arsenal of poplars [46]–[49], negative effects on biotic interactions with EM fungi may be anticipated in transgenic trees with changed lignin biosynthesis. Although we found differences in the EM community composition in the second year after planting, these differences could not be related to the suppression of CCR, CAD or COMT.

The composition of EM communities can be influenced by abiotic and biotic environmental factors such as fungal competition [50], soil nutrient and water availability [51]–[53] and the physiology and genetic constitution of the host [34], [54], [55]. Variations of abiotic factors and patchiness of soil fungi were not detected in our study plantation. Therefore, EM species composition and abundance might have been influenced by host factors. During transformation the positioning of the introduced DNA in the genome cannot be controlled. Thus, the insertion may have side-effects when the introduced DNA fragment unintentionally hits a functional plant gene locus. Therefore, each transformation event may cause intra-specific variation of traits, in addition to the target gene. Controlled experiments testing the colonization efficiency of the EM fungus *Laccaria bicolor* with the F1 progeny of an inter-specific poplar hybrid revealed that the ability to form mycorrhizas underlies natural intra-specific variation [55]–[57]. Different EM assemblages were also observed in the present study for different varieties of *P*. x *euramericana*, a poplar hybrid bred for biomass plantations [58], [59]. The intra-specific and inter-specific variation in EM assemblages on the WT hybrids of *P*. x *euramericana* and *P*. x *canescens* was similar to that between CCR line L7 and CAD line 22, which exhibited the largest difference of EM species composition. Our study, therefore, supports that the host genotype can affect the colonization ability of distinct mycorrhizal fugal species. However, the intra-specific variation introduced by the transformation of poplars with the antisense constructs to suppress CCR, COMT or CAD activities did not result in larger differences in the EM community composition than those observed for different varieties of conventionally bred high-yielding poplar clones.

### The link between EM colonization and diversity and poplar dendromass and nutrient status

The GM poplars with suppressed activities of enzymes of lignin biosynthesis showed strong (ca. 5-fold) differences in growth and biomass in the plantation. This was not surprising since similar results had been obtained by others studying the performance of lignin-modified plants. For example, Leplé *et al*. [15] found reduced growth in two of five investigated CCR-suppressed poplar lines under field conditions. Voelker *et al*. [60] observed extensive variations in aboveground biomass of 14 different lines of *P. × canescens* down-regulated in 4-coumarate:coenzyme A ligase (4CL). Furthermore, greenhouse-grown transgenic poplars with suppressed coumaroyl 3′-hydrolase (C3′H) activity showed drastic growth reductions [61]. The suppression of C3′H activity also reduced the water use efficiency resulting in lower δ^13^C signatures in the transgenic compared to WT poplars [61]. If the growth reductions found here were due to impairment of photosynthesis such as reduced stomatal conductance, we would have expected a shift in the δ^13^C signature to higher values because of decreased carbon discrimination. However, this was not observed and, therefore, effects on water use and carbon allocation to wood are unlikely reasons for growth reductions in the GM poplars of our study.

Another possibility is that changes in EM colonization and changes in the EM communities had negative impact on tree nutrition leading to reduced growth. This option is not unlikely since the interactions of mycorrhizas with their hosts cover the whole range from beneficial to parasitic effects [62], [63]. For example, colonization of *P*. x *euramericana* (cv Ghoy) with different arbuscular mycorrhizal fungal species caused reductions in plant biomass [64]. Although the P concentrations of the aboveground tissues increased, P content of the shoot was diminished because of overall biomass loss [64]. In our study, the abundance of the EM fungi *Peziza ostracoderma* and the ascomycete JQ JQ409294 on root tips of the transgenic poplar genotypes showed negative and positive correlations with foliar P concentrations, respectively. *Paxillus involutus*, which was present in our plantation, has been shown to increase K and P nutrition of poplars [20]–[23], [65]. These observations might imply that distinct EM-poplar genotype associations contributed to facilitating or suppressing P or K transfer to their host trees. However, this suggestion is currently speculative since a full nutrient budget of the trees was not possible and the regulation of tree-fungal-environmental interaction is barely understood. Further functional analyses of EM fungi are, therefore, required.

N is one of the most important nutrient elements for plant growth [66]. In young strongly growing poplars N is mainly present in leaves, but a significant fraction is resorbed in fall, present in woody tissues during the dormant season and re-utilized for sprouting in spring [67], [68]. Here, we observed a negative relationship between stem N concentrations and stem biomass indicating higher storage in the wood of smaller poplars than in those of taller plants. The biomass differences of stems were maintained in the following season, and could obviously not be compensated by increased internal N utilization of smaller trees for stem growth. Thus, poplars with low growth have the additional disadvantage of wasting N when utilizing woody biomass. There is evidence that N allocation differs between fast and slow growing poplar species since trees with inherently higher biomass production exhibit lower N concentrations in the wood and higher nitrogen productivity [69]–[71]. Poplars grown on a previous agricultural field also showed increased biomass production, decreased N concentrations, and increased nitrogen use efficiency in response to long-term free air CO_2_ enrichment [72], [73]. Our present data support that, at least in the initial phase, EM colonization is linked with these traits. Positive relationships for growth, nitrogen utilization and EM colonization rates have also been found in Douglas fir [74]. Based on the current data it is not possible to distinguish if poplar growth was stimulated because of higher rates of EM colonization or if trees with higher growth were more amenable to EM colonization. However, the latter possibility is more likely since other studies have already shown that EM colonization and diversity were driven by carbon availability and productivity of the host tree and not vice versa [34], [54], [74]. Since the root tips of the GM poplars were almost completely colonized with EM at the end of the second growing season, it is clear that the GLM model developed for biomass, nitrogen and root colonization will not be applicable in older plantations. The establishment phase is, however, very important and biomass increments realized during this crucial period will result in further gains because of the exponential nature of growth.

## Conclusion

Genetically modified poplars are a potential alternative for the production of renewable energy since their properties can be optimized to facilitate saccharification. The release of transgenic organisms into the field needs to be carefully controlled to avoid negative effects on environmental interactions, especially with potentially beneficial soil microbes. In this study we demonstrated that transgenic poplar lines modified in the lignin biosynthesis pathway show normal abilities to form ectomycorrhizas. Gene-specific effects of the transformed poplars on mycorrhizal community structure were not found. Variations in EM community structures found between different GM poplar genotypes were in a range similar to the intra-specific variation of commercial poplar clones. The transgenic lines displayed strong differences in stem biomass production. Wood production in the initial phase of plantation establishment was positively correlated with EM colonization rates and negatively with stem N concentrations. Growth advantages realized in the establishment phase were pertained in the following year. Our results suggest that initial differences in EM colonization may have consequences for long term biomass production.

## Supporting Information

Figure S1 **Overview of the experimental plantation of** ***Populus*** **x** ***canescens*** **.**
(DOCX)

Figure S2 **Overview of the commercial plantation of** ***Populus deltoides × P. nigra*** **.**
(DOC)

Table S1 **Dissimilarity matrix of fungal communities based on the DGGE band pattern.**
(XLS)

Table S2 **Soluble amino acid, nitrate and ammonium concentrations in soil samples collected in 2008.**
(XLS)

Table S3 **Relative abundance of fungal species detected on ectomycorrhizal root tips of** ***P. × canescens*** **and** ***P. deltoides × nigra*** **by morphotyping/ITS-sequencing.**
(XLS)

Table S4 **Diversity indices of ectomycorrhizal fungal communities on the roots of P. × canescens in 2009 and 2010.**
(XLS)

Table S5 **Mean nutrient element concentrations in leaves, stem, and roots of wildtype and transgenic poplar (** ***P. × canescens*** **) genotypes.**
(XLS)

Table S6 **Pearson product moment correlations between biomass, ectomycorrhiza and nutrient related parameters.**
(XLS)
