# Supplementary material for: Ectomycorrhizal Colonization and Diversity in Relation to Tree Biomass and Nutrition in a Plantation of Transgenic Poplars with Modified Lignin Biosynthesis
Source: PLoS One. 2013 Mar 13;8(3):e59207. doi: 10.1371/journal.pone.0059207 (PMC3596300; doi:10.1371/journal.pone.0059207)
Supplement: Figure S1 — Overview of the experimental plantation of Populus x canescens . (DOCX) [file pone.0059207.s001.docx]

**Fig. S1: Overview of the experimental plantation of *Populus* x *canescens*.**

The experimental field covered an area of 1365 m^2^. In total 120 plants per poplar line were planted in a randomized block design. Each block consist 8 subplots one for each poplar line. Each subplot consists of 24 trees planted in two double rows with 6 trees in each row. The space between trees of one double row was 0.55 m while the interspace between the two double rows was 1.5 m, planting distance within one row was 0.5 m. Different transgenic lines are labeled by different abbreviations (WT, CCR: L5 and L7, COMT: L9 and L11, CAD: L18, L21, and L22). To prevent an edge effect the field was bordered with one row of wildtype clones (not shown). Sampling location in 2008 (black pots), 2009 and 2010 (both grey square) are labeled in the figure.

**
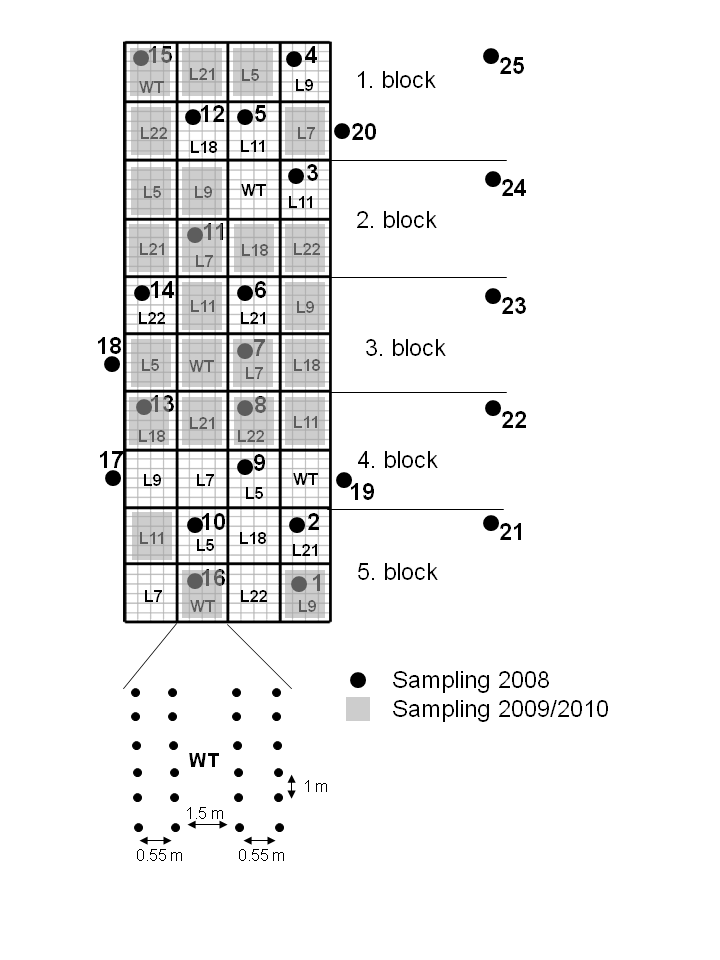
**
