# Supplementary material for: Ectomycorrhizal Colonization and Diversity in Relation to Tree Biomass and Nutrition in a Plantation of Transgenic Poplars with Modified Lignin Biosynthesis
Source: PLoS One. 2013 Mar 13;8(3):e59207. doi: 10.1371/journal.pone.0059207 (PMC3596300; doi:10.1371/journal.pone.0059207)

**Fig.S2: Overview of the commercial plantation of *Populus deltoides × P. nigra***

Commercial clones were planted in a randomized block design. Each of the three blocks consist of 11 subplots one for each clone. Each subplot consists of 16 trees planted in four rows with 4 trees in each row. The space between trees of one double row was 0.6 m while the interspace between the two double rows was 1.5 m, planting distance within one row was 0.6 m. Different commercial clones are labeled by different abbreviations (S: Soligo, G: Ghoy, I: I-214, R: Robusta, L: Lambro, K: Koster, F: Flevo, T: Triplo, B:Blanc de Poitou, C: Carpaccio, D: Dorskamp ). To prevent an edge effect the field was bordered with two rows of wildtype clones (not shown). Sampling location for in 2010 for Ghoy, Soligo and I-214 are indicated by a grey square.


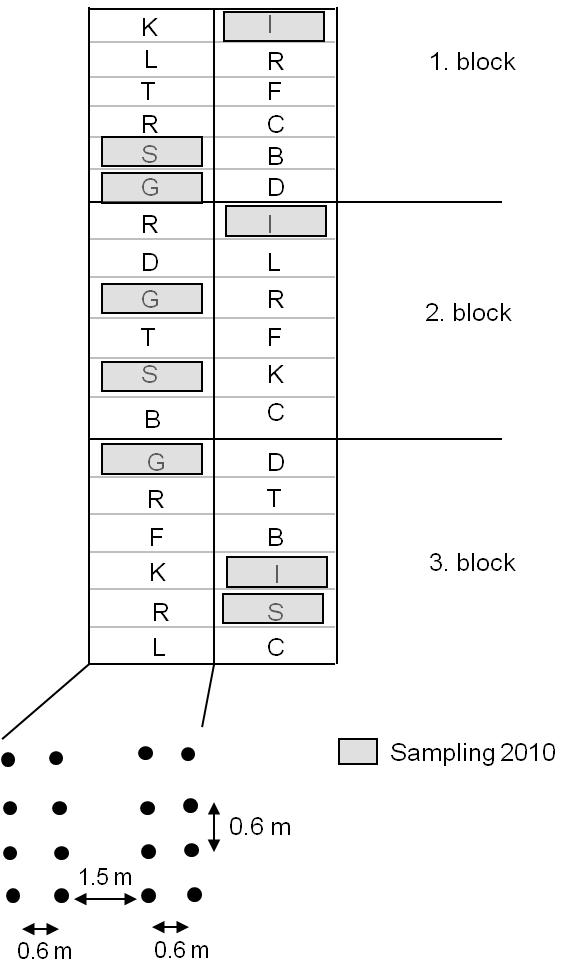

Supplement: Figure S2 — Overview of the commercial plantation of Populus deltoides × P. nigra . (DOC) [file pone.0059207.s002.doc]
